# Implementing an initiative to promote evidence-informed practice: part 1 — a description of the Evidence Rounds programme

**DOI:** 10.1186/s12909-019-1489-y

**Published:** 2019-03-06

**Authors:** Aislinn Conway, Maura Dowling, Áine Binchy, Jane Grosvenor, Margaret Coohill, Deirdre Naughton, Jean James, Declan Devane

**Affiliations:** 1 Health Research Board Trials Methodology Research Network, Galway, Ireland; 2 School of Nursing and Midwifery, National University of Ireland Galway, Galway, Ireland; 3 St. Clare’s Neonatal Intensive Care Unit, Galway University Hospitals, Galway, Ireland; 4 University Hospital Galway, Galway University Hospitals, Galway, Ireland

**Keywords:** Health services research, Education, Dissemination, Implementation, Knowledge translation, Evidence-informed practice, Context, Adaptation, Sustainability, TIDieR

## Abstract

**Background:**

Evidence-informed practice is fundamental to the delivery of high quality health care. Delays and gaps in the translation of research into practice can impact negatively on patient care. Previous studies have reported that problems facing health care professionals such as information overload, underdeveloped critical appraisal skills, lack of time and other individual, organisational and system-level contextual factors are barriers to the uptake of evidence. Health services research in this area has been restricted largely to the evaluation of program outcomes. This paper aims to describe the implementation process of an educational initiative for health care professionals working in midwifery, neonatology or obstetrics aimed at disseminating evidence and enhancing evidence-informed clinical care.

**Methods:**

We designed and implemented an educational initiative called Evidence Rounds for health care professionals working in the women and children’s division of an urban hospital in Ireland. It consisted of three core components: (1) group educational sessions examining evidence on topics chosen by staff (2) a dedicated website and (3) facilitation, enablement and support from a knowledge translation professional. We evaluated user engagement in the educational program by monitoring attendance figures and website analytics. We followed up with staff at 3, 16 and 21-month intervals after the last educational session to find out whether evidence had been implemented. We use Lavis’s organising framework for knowledge transfer and the Template for Intervention Description and Replication (TIDieR) checklist to describe the educational program and document the implementation process.

**Results:**

Six educational sessions presented by 18 health care professionals took place over a nine month period with 148 attendances of which 85 were unique (individuals who attended at least one session). During the period spanning from one month before, during and one month after the running of the group sessions, 188 unique visitors, 331 visits and 862 page views were recorded on our website.

**Conclusions:**

Audit and feedback processes can provide quantitative data to track practice outcomes. Achieving sustainable educational programs can be challenging without dedicated resources such as staffing and funding.

**Electronic supplementary material:**

The online version of this article (10.1186/s12909-019-1489-y) contains supplementary material, which is available to authorized users.

## Background

Evidence-informed practice is central to the delivery of quality care and is associated with improvements in patient outcomes. Emparanza and colleagues [1] demonstrated that mortality and duration of hospital stay figures were reduced among patients treated in an evidence-based practice unit, when compared to either a standard practice unit or previous practice by the same health care professionals (HCPs). Nevertheless, a well-reported gap exists between clinical practice and much of the evidence available to HCPs [2]. When evidence is not translated into practice or there is a delay in the process, patients may be exposed to unnecessary risks or suboptimal care.

There are multiple barriers to evidence uptake and evidence-informed practice. Information overload [3–5] is a barrier which can cause HCPs to become overwhelmed by the volume of available literature when seeking to access the most relevant and up to date research [6]. We have long been in an era of information overload with, for example, more than 1 million publications related to biomedical research captured within the PubMed database each year [7]. Many health care workers have limited time to devote to reading research evidence [2]. Conversely, for some healthcare topics, there can be a lack of evidence or indeed, high quality evidence [8].

There is a need for evidence-informed, theory-based educational and knowledge translation initiatives aimed at HCPs to promote evidence-informed practice and the implementation of evidence where appropriate. There is also a knowledge gap regarding the implementation process of these type of initiatives. One of the most frequently used theories in research looking at the adoption of evidence based practice and implementation science is Everett Rogers’ diffusion of innovations theory (1983). Rogers identified four key elements instrumental to the adoption of an innovation; the innovation itself, communication channels (hereby referred to as modes of delivery), time, and the social system. He categorised stakeholders into five groups according to how they adopt innovations over time; innovators, early adopters, early majority, late majority and laggards [9].

Grimshaw and colleagues [10], highlight that there is a considerable body of evidence relating to KT strategies yet it remains incomplete. A much-debated question is whether combined or single component strategies are more effective [11]. Intuitively, a multicomponent strategy might be more effective when seeking to engage as many clinicians as possible, some of whom may have preferences or circumstances that makes a particular component work for them. However, Squires et al. [12] found that interventions with multiple components were no more effective than single component interventions. They also concluded that the effectiveness of multifaceted interventions did not increase incrementally as the number of components increased. It might be that multiple components used in some studies addressed the same rather than diverse issues or barriers and if so, then this might explain why they were not judged to be more effective. In a systematic review by McCormack et al. [13] multicomponent dissemination strategies focusing on reach, motivation and ability strategies were more likely to affect clinicians’ behaviours than single-component strategies. Another systematic review demonstrated that multifaceted interventions focused on educational meetings to increase implementation of physiotherapy clinical guidelines may improve some outcomes relating to practice but failed to have a positive impact on patient health outcomes or reducing costs [14]. Educational meetings on their own or in combination with other interventions may improve clinical practice or patient outcomes but may not change complex behaviours [15]. A Cochrane systematic review reported that interprofessional education may improve patient outcomes and improve adherence to clinical guidelines although the evidence was judged to be low quality [16]. Wallace and colleagues found that targeted messaging, summaries of research evidence and educational visits may improve the uptake of key research findings [17]. The inclusion of local opinion leaders in an intervention may make it more likely to align HCP behaviours with the desired practice [18]. In a before-and-after study by Segovis, the provision of food was identified by HCPs as a motivating factor to attend grand rounds [19]. According to the National Implementation Research Network (NIRN) based in the United States, an enabling context is an essential component of evidence-based programs for increasing their usefulness [20]. Implementation outcomes and the use of evidence can be driven to a large extent by contextual factors and their methods of delivery [21–23]. Contextual influences on implementation can be both barriers and enablers to different people at different times, under varying circumstances. In a recent systematic review, Geerligs et al. found that barriers and facilitators to implementation processes identified by HCPs were experienced at system, staff and intervention levels [24]. The authors recommend taking these three domains into account when designing implementation strategies. Hamilton and Mittman [21] and Proctor [25] have highlighted the need for further research to describe the implementation of these types of initiatives in sufficient detail.

Informed by this evidence, Evidence Rounds featured a multifaceted strategy centred around educational meetings and focused on increasing the reach of evidence and the motivation and ability to use and apply evidence. We also took an interprofessional approach, by involving multiple professions (midwifery, neonatology and obstetrics) and working with opinion leaders. We designed the initiative to address individual and organizational level factors and adapted it when necessary throughout the implementation process. We arranged for a local catering service to provide food at each session. Our description of the implementation of Evidence Rounds adds to the literature on educational initiatives in applied health services research. There is a general paucity in the existing literature of studies that provide insight into how contextual factors have influenced dissemination and implementation efforts.

Evidence Rounds was based loosely on an intervention conceived by Jacqui Le May, former Head of Knowledge Services at University Hospitals Coventry and Warwickshire, NHS Trust in the United Kingdom (UK). There, members of the Clinical Evidence Based Information Service (CEBIS) team run Evidence in Practice Groups to examine evidence in various departments within the hospital. Topics and questions are linked to a specific patient cases, series of patient cases or other general topics. As well as incorporating the best available evidence into our group sessions, we used evidence from key findings of systematic reviews and other research to inform the design and implementation of the initiative.

The goal of Evidence Rounds was to bridge the gap between evidence and practice through an educational initiative aimed at HCPs. The objectives were to disseminate the best available evidence to HCPs on topics of their choosing during group sessions; to promote evidence-informed practice through the provision of an in-person group platform for staff to discuss the implications of the evidence, the barriers and facilitators to its implementation and, to enhance evidence-informed practice by identifying and assigning resulting actions where appropriate.

The aims of this paper are to describe the process of planning, designing and implementing this multi-component educational initiative, to report data on quantitative performance indicators monitoring engagement during the implementation process and to provide follow up information regarding the implementation or lack of implementation of the evidence. The second paper in this two-part series reports the findings of focus groups and interviews about Evidence Rounds with HCPs who attended or presented at the group educational sessions [26].

## Methods

**Fig. 1:**
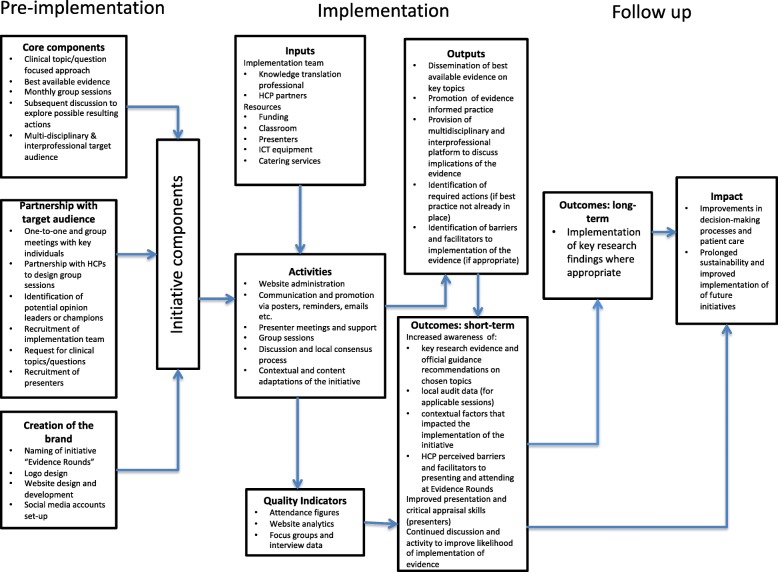
Process-oriented logic model of the Evidence Rounds educational initiative

In Fig. 1, we present a logic model developed iteratively to demonstrate the underlying logic behind the implementation strategy for Evidence Rounds. We designed it with the understanding that implementation processes and health systems are complex. May and colleagues [27] advised that implementation processes be understood as “non-linear, emergent and dynamic events within systems.” The model focuses on the components of the initiative, our planned activities and what we hoped to achieve through the initiative. We informed the pre-implementation and the implementation phases by adapting aspects of the CEBIS Evidence in Practice Groups, Rogers’ diffusion of innovations theory [9], the framework for knowledge transfer [28] and the Knowledge Translation Planning Template [29]. We used Rogers’ diffusion of innovation theory to drive the implementation strategy [9].

The organising framework for knowledge transfer strategies conceived by Lavis et al. [28] was used to develop the implementation strategy. This framework asks five key questions: 1. What should be transferred to decision makers? 2. To whom should research knowledge be transferred? 3. By whom should research knowledge be transferred? 4. How should research knowledge be transferred? 5. With what effect should research knowledge be transferred?

1. *What should be transferred to decision makers?* To improve the likelihood of evidence uptake, HCPs were invited to select topics or clinical questions relating to treatment or diagnostic interventions. A member of staff who later confirmed with colleagues their agreement on her chosen topic suggested the topic for the first group session at a planning meeting. For subsequent sessions, a collective decision was made at group sessions about the topic to be covered in the next session. Sometimes, several suggestions were considered before a decision was made. At the request of one HCP, a topic suggestion sheet was passed around during sessions to accommodate staff who were reluctant to propose topics in front of their colleagues. HCPs were asked to submit suggestions based on gaps they perceived in their knowledge of the evidence or where there was a perceived gap between the evidence and their own practice. Topics were not limited to those known to have clear and conclusive evidence and suggestions covering controversial treatments, those that had conflicting evidence findings, or a lack of evidence, were encouraged. Our aim was to transfer the best available, most up to date, relevant and applicable evidence. A list of sub-questions or topics for each educational session is featured in Table 3. At the start of each session, national and international official guidance was explored to increase awareness of current recommendations. All of the selected topics and clinical questions involved healthcare interventions so we were particularly interested in accessing and presenting randomised trials and systematic reviews of trials. However, for all topics, we also included non-randomised or observational studies so that qualitative aspects of topics could be taken into consideration. For some sessions, HCPs requested and found it valuable to read reports on what other units were doing and compare and contrast their own practice. The final selected topics are presented in Table 3.
2. *To whom should research knowledge be transferred?* Our target audience consisted of HCPs working in the neonatal and obstetric departments in the women and children’s division of an urban hospital in Ireland. We took a multi-disciplinary and interprofessional approach to maximise the potential for the dissemination and implementation of evidence and to promote collaboration with the ultimate goal of implementation of evidence where appropriate. We also invited staff members outside of key departments when deemed appropriate to the topic. For example, laboratory staff were invited to attend the fourth session: antenatal screening for group B streptococcus. When these staff were identified, invitations were extended through the presenting HCPs. The implementation team also invited students who were on placement in the departments during the time of the sessions.
3. *By whom should research knowledge be transferred?* We took a team approach to the transfer of knowledge. Three HCPs presented at each session with representatives from both medical and nursing and midwifery staff in each session. Staff from the neonatal and obstetric departments presented when the topic covered both disciplines. To recruit HCPs to present, staff were asked to volunteer during group sessions or previous presenters contacted individuals they perceived as suitable candidates. The KT professional who is an author on this paper (AC) introduced each session, discussed the literature search process, the breadth of the literature on the chosen topic, and directed discussion to decide on the next topic.
4. *How should research knowledge be transferred?* The KT strategy involved both active and passive methods of promotion, communication and dissemination. In line with Rogers’ diffusion of innovations theory [9], we accepted that our target audience was likely to adopt the evidence presented in the educational initiative at different points in time. Therefore, we deemed it appropriate to use a multifaceted educational strategy. To increase the reach of the evidence: We identified and arranged meetings with key staff at the hospital - to build an implementation team and identify potential champions or opinion leaders that could help us communicate with HCPs and disseminate evidence. Our group sessions targeted multiple disciplines and professions to increase the impact. We employed a variety of communication and dissemination modes of delivery (See Table 1) e.g. face-to-face meetings, telephone calls, emails, an open access website, based on the assumption that we were likely to encounter stakeholder groups similar to those identified by Rogers [9] who may adopt the initiative at different points in the process and for a variety of reasons. To increase motivation to use and apply the evidence: HCPs took ownership by choosing topics that had the potential to improve their practice and that were meaningful and timely for them. We focused on the applicability of the evidence to the local context. When requested, we presented information on how other national and international units were providing healthcare services relating to the topic for benchmarking purposes. In 3 of the 6 sessions, retrospective audit data were presented to capture data relating to recent practice and potentially act as a driving force to change future practice. To increase the ability to use and apply the evidence: We addressed the issue of information overload by designing and performing pragmatic yet comprehensive search strategies, sifting through the frequently large volume of search results and discarding obviously irrelevant records. Searches were ran on appropriate databases and websites including; the Cochrane Library databases, Medline or PubMed, CINAHL, Embase, Google (to identify guidelines and grey literature), relevant professional bodies and organisations’ websites, healthcare organisations’ websites, DynaMed, Trip Database Pro and the Geneva Foundation for Medical Education and Research (GFMER). Presenting HCPs were provided with a significantly reduced number of records to screen for inclusion. After feedback from the first session, a “Quick Guide for Presenters” (see Additional file 1) was provided to HCPs who had signed up to present. Key data and findings from multiple studies were extracted and summarised during group sessions. We fostered an environment where critical appraisal was key and highlighted the strengths and weaknesses of included evidence. The KT professional provided support and enabling services to presenters to reduce their workload and improve levels of health information literacy e.g. obtaining full text of papers, helping with interpreting statistical data e.g. forest plots and key statistical concepts such as *P* values and confidence intervals, identifying appropriate critical appraisal tools, sourcing images to put into presentations (in compliance with licensing and copyright restrictions), providing feedback on presentation slides, populating reference sections, extracting key information and data, providing guidance on selecting papers for inclusion etc. During the discussion forum, obstacles to the implementation of evidence were identified to increase the likelihood that they would be addressed and plans for change could be tailored [30].

**Table 1:** Modes of delivery used in Evidence Rounds for promotional purposes, communication and dissemination

Mode of delivery	Details and contextual influences
Group educational sessions and discussion forum	The presentations at all sessions had a similar structure with small differences if warranted by the topic eg. presenters carried out a retrospective local audit for 3 out of the 6 group sessions. Therefore, repeat attendees became familiar with the format and upcoming and potential presenters knew what to expect. We found that the majority of staff remained behind for facilitated discussion. We promoted an informal and relaxed atmosphere where all disciplines and professions were encouraged to contribute their opinions. At times, it was necessary to refocus discussion on key points related to the topic, to bring the group’s attention to break-off conversations, to encourage discussion of the applicability of evidence to local practice and practical aspects at the hospital that would influence how the evidence would be addressed/handled.
In-person meetings	One-to-one and group meetings were arranged with key informants (eg. practice development and front-line staff interested in research) for implementation planning. These interactions were important for gaining an understanding of the organizational context and choosing the implementation team. It was pivotal to our initiative to gain buy in, and collaborate and partner with HCPs to give them the opportunity to be involved in, contribute to and co-design and development of the initiative. Through recommendations from these meetings and additional contacts, we reached out to those who could be considered as potential opinion leaders and champions. A key intention was to identify people with different professional perspectives to identify their needs and bring them on board. We held meetings with presenters for preparatory, enabling and support purposes. Presenters attended two preparatory meetings, the first after the search strategy was completed to give an overview of results and assign sources and another a few days before the presentation to merge slides, gain clarity about the format of the presentation, make final modifications, summarise information, and identify issues for discussion.
Website	Using a web hosting platform, we designed a logo for Evidence Rounds, purchased a suitable domain name and created a dedicated website. It was designed to present information in a minimalist and aesthetically-pleasing format. During the initiative, the site was updated regularly with current information. The website homepage contained six clickable links, each of which had a distinct core function: • to explain the Evidence Rounds initiative • to act as a repository of presentation slides from group sessions • to provide links to informational resources about searching for, and critically appraising evidence • to present information requested by attendees and presenters. For example, explanations of *p* values and confidence intervals and a brief guide to creating slides for Evidence Rounds group sessions aimed at presenters • to show the schedule of past and future group sessions • to provide contact details for the KT professional We sought informal feedback from staff regarding its usefulness and accessibility. The site was flagged at group sessions, meetings, in email correspondence and on promotional posters (see Additional file 2). When the term “Evidence Rounds” was searched for in the most commonly used search engine, the website did not appear directly and so a desktop shortcut was added to the computer in the neonatal unit. In hindsight, training in search engine optimisation (SEO) which would have been useful to optimise the findability of the website.
Social media	Dedicated accounts on Twitter, Facebook and LinkedIn were set up. After discussion with staff regarding what they and their colleagues were finding useful, it was decided to discontinue updating each of these platforms and concentrate on modes of delivery preferred by staff such as email, word-of-mouth and the website. Staff were keen to manage work-life boundaries when it came to online technologies.
Email	Reminders to attend group sessions were mostly sent via email to staff mailing lists by HCPs from the implementation team. Email was used commonly for communication by the implementation team and presenters and was used to recruit participants for focus groups and interviews. Personalised certificates of attendance or participation (for presenters) were emailed to attendees on an opt-in basis (see Additional file 3 and Additional file 4).
Posters	Staff reported that posters, although a more passive mode of delivery, were effective at reminding them about upcoming group sessions when strategically located. They were designed using an online graphic design service called Canva.
Word of mouth	Word-of-mouth played a vital role in the delivery of information during the implementation process and was deemed a very effective means of engaging our target audience by the implementation team. Some HCPs started attending group sessions based on recommendations from their colleagues and we were told that discussion about evidence covered in group sessions and its implications for practice continued from the classroom to the wards.

At the initial planning meetings, we emphasized that we did not intend on imposing the Evidence in Practice Groups model from the UK on staff at our hospital. Baumann recommends taking an adaptive approach to implementation because no single intervention will be a perfect fit in all settings [31]. Proactive adaptation played a key role in our strategy [32] so that we could shape the initiative in response to important individual, organisational and contextual factors. We tailored it to suit the local context with currently available information before implementation and adapted it iteratively throughout in accordance with feedback loops, observations and performance indicator monitoring. See the Table 2 for a list of core components and some adaptations.

5) *With what effect should research knowledge be transferred?* The main aims of Evidence Rounds were to provide an educational program that disseminated evidence to health care professionals and promoted evidence-informed practice. We undertook process evaluation by capturing and monitoring data for key indicators throughout the initiative. Firstly, we distributed sign-in sheets at group sessions to record attendance figures. We wanted to track neonatal and obstetric staff attendances and identify potential patterns. Secondly, we monitored usage analytics on our dedicated website. Both informed us of the penetration of Evidence Rounds to the HCP community within the department. Thirdly, our focus groups and interviews provided self-reported data on how the HCPs were receiving the educational initiative and how they viewed it in relation to their own evidence-informed practice. Using this data, we identified individual, organisational and intervention level barriers and facilitators to attending and presenting at Evidence Rounds. We were better able to understand the complexity of the behaviours and gauge opinions on whether and how Evidence Rounds was promoting evidence-informed practice for them. These results are published in the second paper of this two part series [26]. Fourthly, we followed up with the implementation team to check the status of evidence implementation. Dissemination strategies play an essential role but on their own, do not guarantee the implementation of evidence [13, 33]. For this reason, and when appropriate during the discussion forum, barriers, facilitators and specific actions to aid implementation of evidence were identified, discussed and actions were assigned to specific HCPs as appropriate. Three months after the final group session, we followed up with HCPs on the implementation team to see whether Evidence Rounds had influenced practice. They reported that a small number of recommendations from Evidence Rounds had been implemented. When implementation happens, the process can be slow, particularly for more complex issues. In the interviews and focus groups, several HCPs explained that changes in practice often cannot occur until the desired change is firstly made a part of a clinical guideline [26]. Writing and updating guidelines can be a lengthy process. Further follow up with the same HCPs occurred 16 and 21 months later.

**Table 2:** TIDieR checklist

Item number	Item	Description
Brief name		
1.	Provide the name or a phrase that describes the intervention.	Evidence Rounds
Why		
2.	Describe any rationale, theory, or goal of the elements essential to the intervention.	This information is provided in the Background section of this paper.
What		
3.	Materials: Describe any physical or informational materials used in the intervention, including those provided to participants or used in intervention delivery or in training of intervention providers. Provide information on where the materials can be accessed (e.g. online appendix, URL).	Physical materials: • laptop, projector, cables, presenter remote, printer, paper, ink cartridges • audio recording equipment, extension lead Informational materials: • Evidence Rounds website: www.evidencerounds.com. See Table 1 for more details. • Posters promoting educational session (see Additional file 2), participant recruitment posters, signage • participant information leaflets and consent forms for focus groups and interviews (reported in part 2 [26]) A budget contributed towards facilitator costs, catering services, printing, website development and hosting services
4.	Procedures: Describe each of the procedures, activities, and/or processes used in the intervention, including any enabling or support activities.	1. *Selection of clinical question or topic* – HCPs invited to submit and when necessary, gain consensus on suggestions 2. *Recruitment of staff to present* - 3 HCPs presented evidence at each session. Staff either volunteered or members of the implementation team contacted specific staff to invite them to present based on their area of expertise 3. *Search for evidence and screening* - One of the researchers (AC) performed focused literature searches and initial sifting of obviously irrelevant results. The HCPs who were presenting the session in question, each screened the remaining results to narrow it down to the resources which they judged to be the best available evidence or key official guidance on the topic. Each resource was considered in terms of relevance, level of evidence and currency. *4. Presentation preparation* • each presenter was assigned records to present according to their preferences of study design and level of experience • presenters used appropriate critical appraisal tools to identify strengths and limitations • presenters decided whether to briefly present local audit data to ground the research and make it more meaningful to attendees • decision about whether topic warranted invitation to HCPs from outside of departments to attend when perceived as advantageous • provision of ongoing enablement and support to presenters e.g. critical appraisal help, extraction and visualisation of data etc. • presenters had the opportunity to plan the final part of their presentation focusing on: o briefly summarising the key findings of the evidence o exploring the relevance and applicability of evidence to local context o identifying potential barriers & facilitators to implementing the evidence *5. Evidence Rounds group sessions* Each monthly session had the following structure: • overview of official guidance and summarisation and critical appraisal of key evidence • discussion of relevance and applicability of evidence to local practice • if applicable, identification of potential barriers and facilitators to implementation of evidence • if applicable, discussion and decision making regarding actions to be taken in light of the evidence and assignment of actions to responsible persons
Who provided		
5.	For each category of intervention provider (e.g. psychologist, nursing assistant), describe their expertise, background and any specific training given.	Presenters and other members of the implementation team were qualified physicians, nurses or midwives. The initiative was led by a knowledge translation specialist who has experience of collaborating with HCPs to promote evidence-informed practice in women and children’s divisions at hospitals and had the following training: • Postgraduate Diploma in Research Methods in Health Sciences (University of Warwick, UK) • Knowledge Translation Professional Certificate (St. Michael’s Hospital Toronto and the University of Toronto, Canada)
How		
6.	Describe the modes of delivery (e.g. face-to-face or by some other mechanism, such as internet or telephone) of the intervention and whether it was provided individually or in a group.	The modes of delivery are described in Table 1.
Where		
7.	Describe the type(s) of location(s) where the intervention occurred, including any necessary infrastructure or relevant features.	Each educational session was delivered in a classroom located adjacent to wards for the convenience of staff who could be bleeped or called away at any moment. After the second session, we discussed the possibility of changing to a larger venue but decided against this as the location worked well and the capacity it held was viewed as ideal for promoting discussion, had adequate seating capacity and audio-visual technology to display presentation slides. Interviews and focus groups took place in the maternity boardroom, maternity classroom or in HCP offices within the department. A few preparatory meetings for presenters took place in the hospital canteen or a nearby café to align with staff lunchtimes.
When and How much		
8.	Describe the number of times the intervention was delivered and over what period of time including the number of sessions, their schedule, and their duration, intensity or dose.	Six educational sessions were delivered over nine months. We took a flexible approach to scheduling by avoided exam times, holidays, training or other educational sessions and meetings in order to maximise attendance figures. As requested by staff, Evidence Rounds group sessions were scheduled during lunchtimes. Each session lasted approximately 1 h because the implementation team identified this as a realistic duration of attendance for most HCPs. Immediately after each educational presentation, a facilitated discussion forum took place which lasted for up to 30 min. They were timetabled on Fridays at lunchtimes (excluding the final session which took place on a Wednesday). Before each session email reminders were sent to potential attendees by HCP members of the implementation team and in some cases they delivered in-person reminders on the hospital wards.
Tailoring		
9.	If the intervention was planned to be personalised, titrated or adapted, then describe what, why, when, and how.	Evidence Rounds was adapted throughout its duration in response to the needs and expressed preferences of the audience and the local context, as was planned. All feedback from attendees was considered and acted upon if appropriate and possible, at the earliest opportunity so that subsequent delivery was improved.
Modifications		
10.	If the intervention was modified during the course of the study, describe the changes (what, why, when, and how).	The initiative was modified throughout the course of the study in accordance with feedback from users and observations. For example: • specific patient cases were not a formal part of the presentation • local audit data was collected retrospectively and reported at 3 of the 6 sessions • a brief “Quick guide for presenters” was uploaded to the website in response to frequently asked questions (see Additional file 1) • social network accounts on Facebook, Twitter and LinkedIn were abandoned due to lack of interest. Additional effort was put into posters, website information, email correspondence and face-to-face interactions as preferred by HCPs • the schedule of group sessions was altered to accommodate staff holidays, exams and other educational events to optimise attendance. Therefore, the initiative was delivered over 9 rather than the original plan of 6 months • certificates of participation and attendance were introduced in response to a request from staff after the second session • in the final group session, one of the presenters was not working as a HCP at the hospital. He is the author of 2 papers that were going to be discussed so he was identified as the best person to present the findings. He is an author on this paper (DD) • during one of the group sessions, an attendee requested the circulation of a topic suggestion sheet so that individuals who for whatever reason did not want to make suggestions in front of their colleagues, could contribute.
How well		
11.	Planned: If intervention adherence or fidelity was assessed, describe how and by whom, and if any strategies were used to maintain or improve fidelity, describe them.	We did not assess adherence or fidelity. However, core components of the initiative were identified before the first session and adhered to throughout the duration. Those components were: 1. Clinical question or topic -focused approach to deciding on the content of educational program 2. Literature searches to be conducted by experienced professional 3. Aim to include the best available evidence 4. Monthly group sessions 5. Discussion forum after presentations to discuss possibility of and identify resulting actions 6. Multidisciplinary and interprofessional target audience
12.^a^	Actual: If intervention adherence or fidelity was assessed, describe the extent to which the intervention was delivered as planned.	N/A

^a^This item is not applicable for the intervention being described

We took measures to plan for sustainability (continuation of the initiative after support from the KT Specialist ended) such as developing tools that could be handed over easily. For example, we chose a web hosting platform that allowed us to build the website and create content using high quality templates without the need for coding or programming skills. Our choice was deemed the most likely option to promote sustainability because at the end of the period of support from the KT specialist, it could easily been handed over to a HCP lacking advanced technical skills of website design and administration/maintenance. We also linked in with library staff to confirm that they would be willing to design and conduct future searches, had conversations with key people, discussed it during our focus groups and interviews and offered guidance during a handover period. We planned to assess sustainability by following up with the implementation team to find out whether the educational initiative had continued to be delivered.

We employed the Template for Intervention Description and Replication (TIDieR) checklist, to complement the reporting of the initiative [34]. This reporting guideline has been recommended for use to report intervention implementation [35].

We collected and report a number of quantitative measures:

- website analytics captured by our website hosting platform. We report figures spanning the period from one month before the first group session, during the group sessions and one month after the last group session:

1. *unique visitors* defined as the number of visitors visiting for the first time
2. *visits* defined as the number of browsing sessions and can involve multiple page views
3. *page views* defined as the number of times a webpage from our website was fully loaded by a browser

- the total number of HCPs and other attendees who attended each Evidence Round session (other attendees included academic partners and students from health-related higher education courses on placement at the hospital site)
- the total number of HCPs who presented at an Evidence Rounds session.

We contacted the 5 HCP members of the implementation team three, 16 and 21 months after the initiative ended to find out whether Evidence Rounds had led to the implementation of research findings.

## Results

Six Evidence Rounds group sessions were run over a 9-month period. There was a total of 148 attendees of which 85 were unique (individuals who signed the attendance sheet at a minimum of one session). See Table 3 for a breakdown of attendance numbers by educational session. Attendance numbers fluctuated according to factors such as the chosen topic (some of which were common to midwifery, neonatology and obstetrics, and some of which were primarily neonatology-focused), level of interest in the topic subject matter and clinical staffing levels.

**Table 3:** Evidence Rounds session details and follow-up

Session number, topic and (number of attendees who signed in)	Specific questions/ issues explored	Potential resulting actions identified	Resulting actions and contextual factors
1. Premedication for non-emergency neonatal intubations (17)	• What are the risks and benefits of using premedication for neonatal intubation? • What are the risks and benefits of not using premedication? • What are the most safe and effective premedications to use? • What is the current practice in other units (national & international)?	• Develop a policy for premedication for non-emergency neonatal intubation. • It should recommend the following medications: o Administer remifentanil or fentanyl instead of morphine as it has a more rapid onset and a shorter duration of action o Administer suxamethonium instead of pancurionium o Add atropine a preventative, vagolytic agent to prevent bradycardia during intubation • Arrange with pharmacy to stock medications • Introduce colour-coded sticky labels to assist staff in ensuring that medications are offered in the correct sequence • Arrange staff training • Audit practice	Evidence Rounds identified as the ‘driving force’ for the policy. The medical recommendations were added as an appendix to the neonatal intubation policy and all staff are required to confirm that they have read and understand the policy. Colour-coded labels have been introduced. While there is agreement for the need to audit practice, elective intubation is infrequently performed so an audit of practice has not yet been completed. When it does happen, there are plans to audit elements of each intubation.
2. Timing of umbilical cord clamping (32)	• The impact on delayed resuscitation at delivery • Should resuscitation begin with the baby still attached to the cord? • What do the current guidelines say? • Benefits and risks to term and preterm infants • Obstetric implications for the mother	• Change discharge sheet to include optimal timing of cord clamping. • Offer delayed cord clamping (DCC) to preterm infants in addition to term infants which has already been the case. • Conduct audit to assure compliance with documentation	Staff report a ‘concerted effort’ to offer DCC to preterm infants since Evidence Rounds educational initiative. Audit conducted - 8 out of 11 babies ≤35/40 at birth were documented as having received DCC from between 30 to 60 s. Staff report plan to audit preterm infants < 35 weeks every 3 months.
3. Medical management of patent ductus arteriosus (PDA) in preterm infants (20)	• What are the risks and benefits of using medical treatments (specifically indomethacin, paracetamol, ibuprofen) for treating PDA in preterm infants? • What are the risks and benefits of not using them in this population?	Confirmation that best practice was currently in place which is not to routinely treat asymptomatic cases of PDA. Create a standard operating procedure (SOP) for management of PDA particularly for junior doctors who frequently rotate into the neonatal intensive care unit (NICU).	In December 2018, a doctor was writing this standard operating procedure using evidence presented during the educational session. The same doctor was reported to be planning an audit of practice.
4. Antenatal screening for group B Streptococcus (GBS) (32)	• What is the rate of recurrence of GBS? • What is the optimal timing for screening? General thinking = 35–37 weeks • What are the long term effects on infants who have been treated with antibiotics for GBS? • Should women with prolonged SROMs at term (of unknown GBS status) be offered screening? • Should women be offered a patient information leaflet?	The evidence presented at this educational session highlighted a) the increased risk of early-onset group B Streptococcus (EOGBS) in infants of women with risk factors and b) the existence of strategies (screening or intrapartum antibiotic prophylaxis (IAP)) that could reduce the risk. There was consensus amongst staff that there was a need for action because women with GBS in a previous pregnancy were not being offered either strategy. The recommendations from this session were to offer screening to all women who had GBS in a previous pregnancy and to change the local guideline accordingly. Audit patient charts regularly.	After this educational session, the Royal College of Obstetricians and Gynaecologists (RCOG) published their Green-top Guideline no.36 Prevention of Early-onset Neonatal Group B Streptococcal Disease [36]. A staff decision was made to follow the RCOG guidance to screen, however the culture medium to screen was not available at the hospital. Therefore, the local guideline was updated to recommend that all pregnant women who had GBS in a previous pregnancy be informed of their increased risk and offered IAP. In this example, the recommendation from Evidence Rounds was not implemented due to an organisational barrier i.e. a lack of screening medium. Nonetheless, Evidence Rounds increased staff awareness of research evidence and local audit data, promoted discussion and increased motivation to change the guideline and clinical practice. Audit of 10 patient charts each month have confirmed high levels of compliance with change in practice
5. Antenatal steroid use for preterm deliveries less than 37 weeks gestational age (GA) (20)	• At what GA should the corticosteroid be administered? • Identifying mothers at risk. • Routine administration to twins or triplets • When should steroids be repeated?	The consultant dealing with the patient should consider antenatal steroids when there is a risk of preterm birth at a gestational age of 23 weeks + 0 days to 23 weeks + 6 days (previously 24 weeks + 0 days). Change guideline on preterm premature rupture of the membranes (PPROM) to reflect this.	There was a gap in knowledge of the evidence prior to Evidence Rounds. After the educational session, awareness of the evidence increased and it was discussed at subsequent meetings. The local guideline was updated and practice changed.
6. Fetal blood sampling (FBS) (27)	• The specificity and sensitivity of FBS. • Does FBS have any impact on C-sections and instrumental delivery rates? • Is taking a sample from the fetal scalp a true reflection of fetal well-being? • The differences between the FIGO and NICE guidelines in interpreting CTG’s and criteria for FBS. • Normal pH levels of the baby during labour • FBS in presence of Meconium • FBS in reducing incidence of HIE/Cerebral palsy. • CTG monitoring with FBS vs. CTG only without FBS	The evidence presented in this session demonstrated that digital fetal scalp stimulation is effective as a first option in fetal monitoring if a cardiotocography (CTG) trace is pathological. If the fetal heart rate accelerates, the FBS should only be undertaken if the CTG trace is still pathological. This means that FBS procedures, which are more invasive for mother and fetus, will be reduced. Staff to update existing fetal monitoring guideline accordingly.	The local guideline was updated to reflect these recommendations. The implementation team reported an increased awareness of the evidence however, there has been no real practice change. Staff are questioning why, there are education sessions every month and this topic is frequently discussed at caesarean section meetings.

Seventeen HCPs who worked at the hospital presented during the period of implementation. One external HCP (DD, who is an author of this paper), was asked to present at a session because he authored two relevant papers that were selected for inclusion in the presentation (session number 6).

**Fig. 2:**
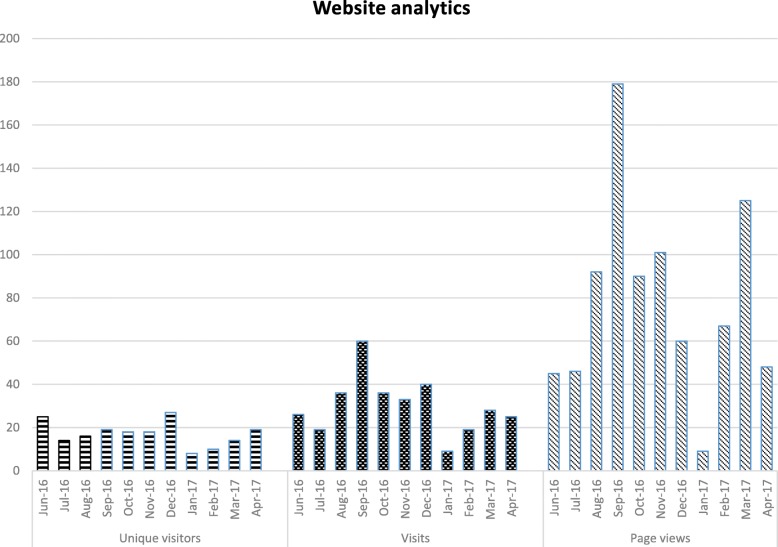
Website analytics data showing number of unique visitors to the website, visits (number of browsing sessions) and number of page views (requests on the website which were fully loaded), by month and year

Between 01/06/2016 and 29/04/2017, 188 unique visitors, 331 visits and 862 page views were recorded on the website. See Fig. 2 for a breakdown of these figures.

In Table 3, we present the clinical questions and topics explored, the resulting actions identified during the discussion forum and the actual resulting actions that were carried out for each of the 6 educational sessions. This information was gathered during follow up with the implementation team.

Follow up with the implementation team also confirmed that the educational program was not sustained beyond the period of support from the KT Specialist.

## Discussion

### Limitations and lessons learned

We would like to acknowledge that our study has several limitations. Firstly, the six educational sessions were carried out over nine months. It is unlikely that this was a sufficient duration of implementation to allow for the initiative to realise its full potential, become fully integrated or adopted by staff that Rogers [9] might describe as the late majority and laggards. In this way, the potential of Evidence Rounds to demonstrate sustainability may have been restricted. Secondly, our theoretical approach did not include pedagogical theory to develop our educational initiative. Thirdly, attendance data collected through sign-in sheets can be viewed as a conservative estimate of actual attendance figures. We are aware of several attendees who did not sign in during sessions for reasons such as being bleeped or called away to attend to a patient. Fourthly, the number of unique visitors recorded using website analytics may be inaccurate because the same person could potentially access the website multiple times using more than one IP address or computer. This would have resulted in them being counted as more than one user. Fifthly, our initiative was implemented at one institution and may be received differently by HCPs in other settings. Sixthly, the information presented in Table 3 regarding follow up lacks quantitative data measures of practice changes following the educational sessions, compared to prior practice. The study by Emparanza [1] provides a good example of quantitative outcome measurement.

In terms of implications for practice, the issue of sustainability is important to consider. Despite the steps we describe in the Methods section aimed at increasing the sustainability of the initiative, it was not sustained beyond the period of support from the KT professional. Without a nominated person or team with dedicated professional hours and taking into consideration the time spent planning and developing, we were aware that there was reduced potential to sustain the initiative at our busy hospital setting. Ideally, future initiatives will have a longer period of implementation to allow for appropriate capacity building and so that they have a better chance of integration and becoming accepted and adopted by staff.

A key learning point for us has been that initiatives like Evidence Rounds are only as strong as the people involved. We recommend collaboration and partnership with the target audience starting from the planning stages and continuing throughout. The multi-disciplinary and interprofessional approach worked very successfully for Evidence Rounds and according to informal feedback and our focus group and interview data [26] it was highly valued by our target audience. We engaged with them, listened to their feedback and found ways to address their identified needs when possible. Our key message in this regard would be to network and engage with champions, opinion leaders, enthusiastic individuals, early adopters and do not wait around for *laggards*. Involving an Information Specialist or Librarian or someone who has knowledge of appropriate databases and other online resources and is experienced in carrying out systematic and detailed literature searches is essential. They can help to address issues of information overload and reduce the workload of HCPs involved in presenting.

Adaptation and adherence to a small number of core components was a fundamental of the initiative. Baker et al. [37], found that positive outcomes are more likely if an adaptive approach is taken to implementing interventions when compared to no intervention or dissemination alone. Feedback from HCPs who participated in our focus groups and interviews suggested that choosing topics based on when guidelines are being created or updated increases the likelihood of implementation of evidence.

Further studies are required to assess the effectiveness of Evidence Rounds, similar educational initiatives including those implemented in settings in the developing world. Evaluation could include pre and post-testing of knowledge of topics the initiative addressed, impact on HCP behaviour and patient care outcomes. More studies are needed to better understand and identify additional underlying mechanisms and contextual factors that influence educational programs. Additional research is also needed to understand how a social media strategy might be optimised for use in the delivery of similar initiatives.

## Conclusion

Evidence Rounds presents a novel educational initiative to support a knowledge translation strategy targeted at HCPs. It moves beyond the journal club model that was familiar to our target audience. It was designed and implemented based on feedback obtained by proactively engaging with staff. We have helped address the need for more research that provides a detailed account of the implementation of knowledge translation strategies [21, 22]. We have also highlighted the contextual factors and modes of delivery that influence implementation outcomes. This paper therefore, will help others to understand the process involved in implementing an educational initiative. Evidence Rounds was a complex initiative to implement due to individual, contextual and intervention-level factors. We used a multi-faceted strategy to disseminate key research findings to our clinical audience and promote evidence-informed practice. We collaborated with and involved our target audience from the start of the planning phase and throughout implementation. This paper provides useful insight into processes and mechanisms involved in rolling out an initiative. We describe the practical aspects or the process of implementing an educational initiative. The level of detail we have provided will aid reproducibility for those wishing to roll out a similar program or elements of the program. We highlighted contextual factors that had an impact on implementation in our setting so that others might use them to inform the planning of their own initiatives.

## Additional files

Additional file 1: Quick guide for presenters (DOCX 59 kb)

Additional file 2: Sample poster promoting Evidence Rounds (PDF 8151 kb)

Additional file 3: Sample certificate of attendance (PDF 103 kb)

Additional file 4: Sample certificate of participation (PDF 103 kb)

## Abbreviations

AB: Áine Binchy
AC: Aislinn Conway
CEBIS: Clinical evidence based information service
CREC: Clinical research ethics committee
DCC: Delayed cord clamping
DD: Declan Devane
DN: Deirdre Naughton
EOGBS: Early-onset group B Streptococcus
GA: Gestational age
GBS: Group B Streptococcus
HCP: Healthcare professional
HRB-TMRN: Health Research Board Trials Methodology Research Network
IAP: Intrapartum antibiotic prophylaxis
JG: Jane Grosvenor
JJ: Jean James
KT: Knowledge translation
MC: Margaret Coohill
MD: Maura Dowling
NCHD: Non-consultant hospital doctor
NICU: Neonatal intensive care unit
NMPDU: Nursing Midwifery Planning and Development Unit
RCOG: Royal College of Obstetricians and Gynaecologists
SOP: Standard operating procedure
