# Supplementary material for: Implementing an initiative to promote evidence-informed practice: part 1 — a description of the Evidence Rounds programme
Source: BMC Med Educ. 2019 Mar 6;19:74. doi: 10.1186/s12909-019-1489-y (PMC6402167; doi:10.1186/s12909-019-1489-y)
Supplement: Supplementary file 1 — Quick guide for presenters (DOCX 59 kb) [file 12909_2019_1489_MOESM1_ESM.docx]

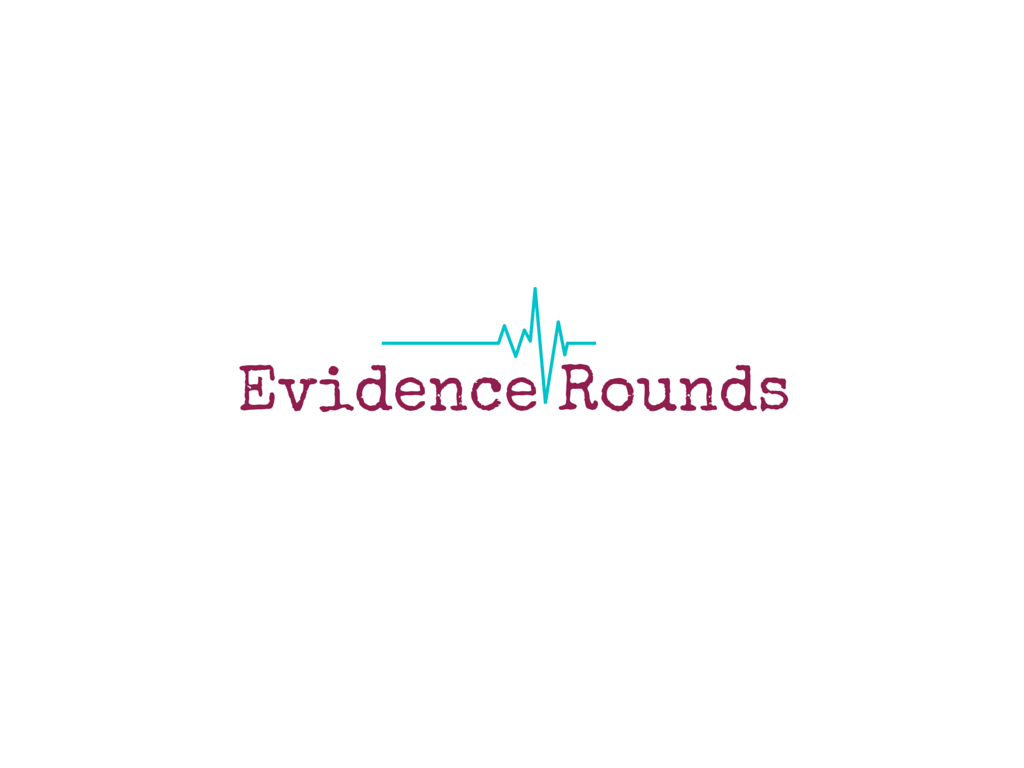


Guidance for Evidence Rounds Presenters

| Checklist of Information to Know about your Chosen Resources* | |
| --- | --- |
| General background information | author, year of publication, country/countries where it took place (if a trial/study) |
| Study design | eg. RCT, systematic review, cohort study etc. |
| Patient/ population/problem | - total number and number in each group/arm - inclusion and exclusion criteria - if systematic review, total number of included studies and total number of study participants across all studies |
| Intervention | dosage, administration, frequency, concurrent patient management that may influence outcomes |
| Comparison(s) | as above |
| Outcomes | both primary and secondary & how they were measured |
| Key findings | in text body and key tables/graphs etc. |
| Strengths of the study/review | Find a critical appraisal tool (suitable for your study design) at the following link: <http://www.evidencerounds.com/resourcesandtools-criticallyappraise> Use it to identify the main strengths and limitations |
| Limitations of the study/review |  |
| Applicability to the local context |  |

*Not applicable to guidance documentation

When Creating your PowerPoint Slides:

- Use a plain white background on all slides
- If using images, please insure that you have the necessary permissions to do so. The finished presentation will be uploaded to the Evidence Rounds website so all copyright and licensing laws must be adhered to. It will be possible to access a bank of images so email [evidencerounds@gmail.com](mailto:evidencerounds@gmail.com) if you are unsure or need help sourcing an image.
- You can insert screenshots of key tables/figures/graphs etc. from the resources. Just make sure to reference them underneath.
- On slides where you are discussing a particular resource, the title of the slide should state the surname of the lead author, *et al* (in italics) if applicable, and year of publication. Example of format: Ohlsson *et al*, 2015.
- On slides where you are discussing a particular resource by a professional organisation, the title of the slide should include the professional body and its acronym in brackets followed by the year. Example of format: Royal College of Obstetricians and Gynaecologists (RCOG), 2014
- Include a reference slide at the end of your section to confirm which resources you have selected and discussed
- Aim for roughly 10-12 slides, you will have approximately 12 mins each to present.
