# Supplementary material for: Implementing an initiative to promote evidence-informed practice: part 1 — a description of the Evidence Rounds programme
Source: BMC Med Educ. 2019 Mar 6;19:74. doi: 10.1186/s12909-019-1489-y (PMC6402167; doi:10.1186/s12909-019-1489-y)
Supplement: Supplementary file 2 — Sample poster promoting Evidence Rounds (PDF 8151 kb) [file 12909_2019_1489_MOESM2_ESM.pdf]

IN THE
**MATERNITY CLASSROOM**

**LUNCH**

PROVIDED BY
MR. WAFFLE

Evidence Rounds

INTERDISCIPLINARY SESSION 6

# FETAL BLOOD SAMPLING

Wednesday

MAR. 29TH
2017

FROM
**12.30-13.30**
ALL STAFF AND
STUDENTS WELCOME

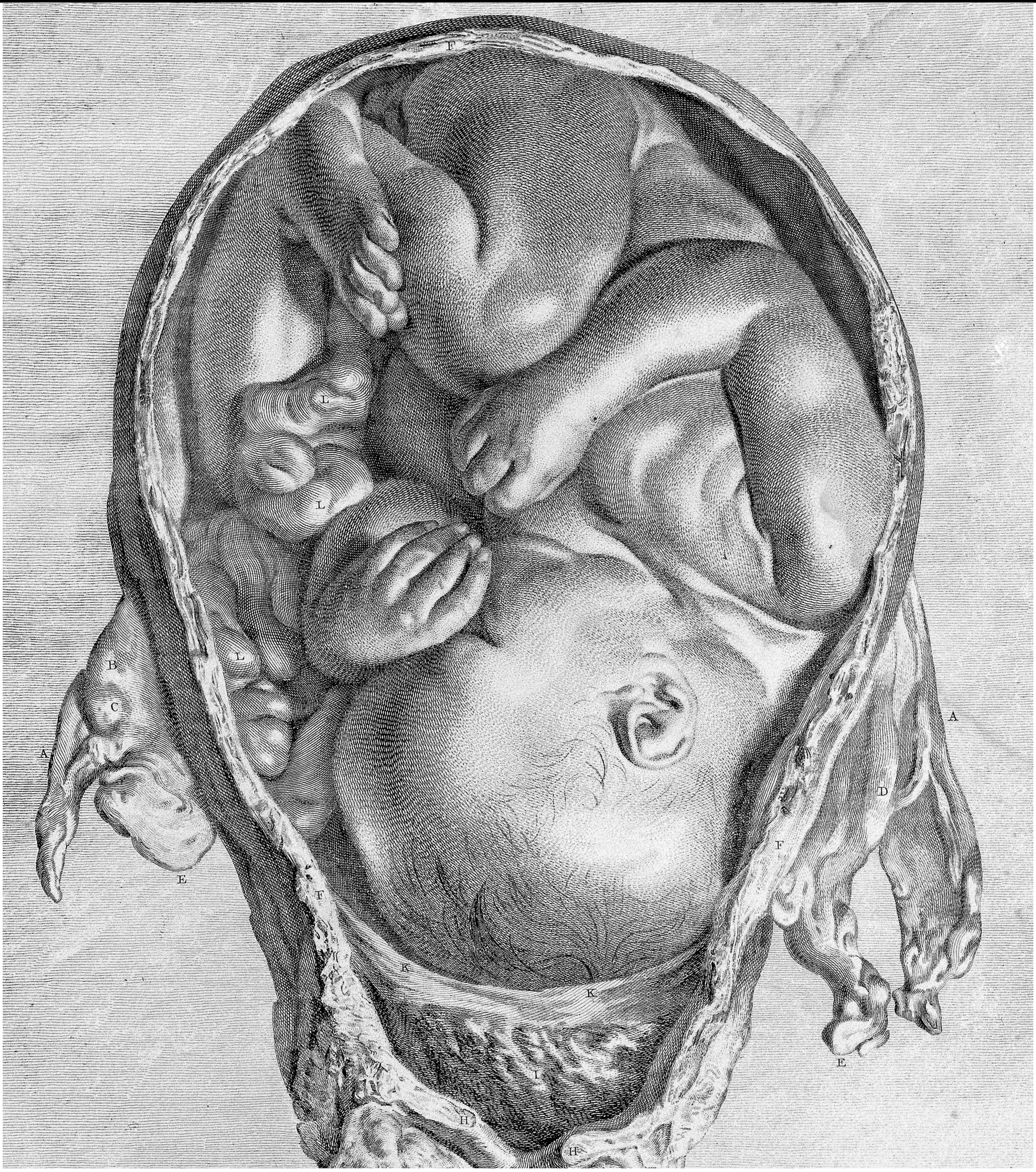

Health Research Board TMRN Trial Methodology Research Network

Saolta
Grúpa Ollscoile Cúram Sláinte
University Health Care Group

OÉ Gaillimh
NUI Galway


WWW.EVIDENCEROUNDS.COM
EVIDENCE ROUNDS@GMAIL.COM
091 123456
