# Supplementary material for: Implementing an initiative to promote evidence-informed practice: part 1 — a description of the Evidence Rounds programme
Source: BMC Med Educ. 2019 Mar 6;19:74. doi: 10.1186/s12909-019-1489-y (PMC6402167; doi:10.1186/s12909-019-1489-y)
Supplement: Supplementary file 4 — Sample certificate of participation (PDF 103 kb) [file 12909_2019_1489_MOESM4_ESM.pdf]

Evidence Rounds

www.evidencerounds.com

CERTIFICATE OF PARTICIPATION

This is awarded to:

Prof. Declan Devane

to certify his participation in
Evidence Rounds 6: Fetal Blood Sampling
at University Hospital Galway
on the 29th of March, 2017.

Aislinn Conway
PhD Fellow, Health Research Board Trials
Methodology Research Network (HRB-TMRN)

Aislinn Conway
Date: 29/03/2017
